# Skin cancer in Germany: Characterising screening, prevalence and mortality from a spatial perspective

**DOI:** 10.1371/journal.pone.0305915

**Published:** 2024-07-05

**Authors:** Jobst Augustin, Valerie Andrees, Matthias Augustin, Nirohshah Trialonis-Suthakharan, Sandra Hischke

**Affiliations:** Institute for Health Services Research in Dermatology and Nursing (IVDP), University Medical Center Hamburg-Eppendorf (UKE), Hamburg, Germany; The University of Sydney, AUSTRALIA

## Abstract

Aim of the study was to characterise the association between screening, prevalence and mortality of skin cancer in Germany considering the spatial distribution. The study included the total set of outpatient data of all statutory health insured people and cause-of-death statistics in Germany between 2011–2015 on county level. To identify regions with high/low screening, prevalence and mortality rates, probability maps were calculated. Scenarios were developed based on the research questions. These were used to identify regions that share both high/low rates of screening, prevalence and mortality. Regression analyses were used to characterise these regions, taking into account sociodemographic characteristics. Significant regional variations in prevalence, screening and mortality in Germany were identified. Depending on the scenario, influences of sociodemographic conditions become apparent. For example, a lower income (p = 0.006) and poorer accessibility of the closest dermatologist (p = 0.03) predicted a lower prevalence of and fewer screenings for skin cancer. In regions with low screening and high mortality, significant (p = 0.03) associations with the educational status of the population were also found. The study identified the first spatial associations between screening, prevalence and mortality of skin cancer in Germany. The results indicate that regional population-related characteristics (e.g., sociodemographic characteristics) play an important role in explaining the associations and should be given more weight in further studies. However, further studies, particularly on the spatial variation of skin cancer mortality, are still necessary.

## Introduction

Skin cancer is the most common cancer worldwide. In skin cancer, a distinction is made between malignant melanoma (MM) and non-melanocytic skin cancer (basal cell carcinoma (BCC) and squamous cell carcinoma (SCC); NMSC). The prevalence and incidence rates have steadily increased in the Caucasian population in the last three decades [1]. Skin cancer carries a substantial impact on quality of life and can be disfiguring and even fatal [2]. However, the impact of living with skin cancer stems also health economic problems and serious public health concerns in high-income countries [2, 3].

Skin cancer, although very common in the fair-skinned population, is preventable through proper behaviour (e.g., protection from UV radiation) and has high chances of cure at an early stage. Accordingly, early detections are therefore crucial, especially in MM. Depending on the country, skin cancer prevention for the population is regulated differently. In Germany, since 2008, a free screening for early skin cancer detection is offered nationwide to people at age 35 and older with statutory health insurance (SHI). This skin cancer screening is performed by a dermatologist or a general practitioner (GP) and can be repeated every two years.

From a spatial perspective, skin cancer prevalence [4], skin cancer screening utilisation [5] and skin cancer mortality [6, 7] are subject to striking variations in Germany. The reasons for this are manifold and can be found primarily in the sociodemographic characteristics of the population, both for prevalence [8] and screening [9].

Most skin cancers should be detected in areas with high screening rates, resulting in high prevalence and low skin cancer mortality (due to early detection). Conversely, where skin cancer mortality is high, screening rates and prevalence should be low. The question is how regions where these hypotheses are confirmed differ from those where they are not. This research project investigates what proportion of regions, in this case counties, confirm our hypotheses and how these can be characterised in terms of socio-economic factors. The aim of this research project is therefore to investigate the relationship between early skin cancer detection (screening), -prevalence and -mortality in Germany. Specifically, the following research questions are addressed:

1. What is the proportion of counties in Germany that have both a high (low) skin cancer *screening* utilisation and a high (low) skin cancer *prevalence*, and how can these be characterised in terms of socio-economic factors?
2. What is the proportion of counties that simultaneously have a high (low) rate of skin cancer *screening* utilisation and a low (high) skin cancer *mortality* in Germany and how can these be characterised in terms of socio-economic factors?
3. What is the proportion of counties that simultaneously have a high (low) skin cancer *prevalence* and a high (low) skin cancer *mortality* in Germany and how can these be characterised in terms of socio-economic factors?

This study is the first attempt to characterise the associations between early skin cancer detection, prevalence and mortality from a spatial perspective. Thus, the study fills a research gap in that it attempts to look at skin cancer screening, prevalence, and mortality in parallel.

## Methodology

### Study design and data

This cross-sectional ecological study was conducted for the years 2011 to 2015. It is based on three data sources: a) nationwide outpatient billing data of the National Association of SHI Physicians (Kassenärztliche Bundesvereinigung, KBV), b) cause-of-death statistics of the statistical offices of the federal states (Statistische Landesämter, StaLa) and c) data on socioeconomic and -demographic characteristics of counties from the Indicators and Maps of Spatial and Urban Development (Indikatoren und Karten zur Raum- und Stadtentwicklung, INKAR) database. A review by the ethics committee was not necessary due to the data used and the study design.

The KBV data set includes about 72.3 million people with SHI, representing about 90% of the German population, and contains for skin cancer screening the EBM (uniform value scale, in German: Einheitlicher Bewertungsmaßstab) codes 01745 (early detection of skin cancer) and 01746 (surcharge for EBM 01732 for early detection of skin cancer). EBM code 01745 is usually billed by the dermatologist, EBM code 01746 by the GP as part of the health check-up 35. Here we have calculated the sum of both EBM codes. The screening rate per district was calculated by dividing the number of screenings per year and county by the number of insured persons in that year and county. Furthermore, this data set provides the data on diagnosis of skin cancer. These includes the ICD-10 codes C43 (malignant melanoma of the skin, MM) and C44 (other malignant neoplasms of the skin, NMSC). Because the screening concerns both the MM and the NMSC, they have been combined here. Another reason for the summary was that the mortality data from the cause of death statistics (source: StaLa) do not distinguish between ICD-10 codes C43 and C44.

Data from INKAR are used to typify the identified regions in terms of socioeconomic and -demographic determinants, such as age, accessibility to the nearest dermatologist or employment rate. In addition to the employment rate, we have also used the unemployment rate. The rationale for this is that the employment rate only covers 75% of all employed persons [10]. Self-employed people, civil servants and other people with a non-employed working status are not included. The selection of variables was literature-based according to Augustin et al. (2020), Akinlotan et al., Vogt et al. and Carsin et al. [5, 9, 11, 12]. The voter turnout was used as a proxy variable for the social situation. For example, Schäfer and Roßdeutscher (2014) show that a high unemployment rate and low education are associated with low voter turnout [13]. Where social problems are concentrated, only half of those eligible to vote turn out even in important elections. The variable “proportion of population reaching the nearest dermatologist within 15 minutes”is based on a geographic network analysis in a Geographic Information System (GIS). Based on a digital street network, physician locations and population distribution, it was possible to calculate the proportion of residents who can reach the nearest dermatologist within a certain time (in this case 15 minutes) by car. Further methodological information can be found in Augustin et al. (2019) [14]. All analyses were performed at county level (N = 402) in Germany (Table 1). A county is an administrative spatial unit that is usually made up of several municipalities, the smallest administrative spatial unit in Germany.

**Table 1:** Overview of used data in this study, structured by data description, variables, entity and source.

Name	Description	Variables	Entity	Year/Period	Source
**Skin cancer screening rates**	Outpatient billing data	ICD-10 C43 (MM)	Proportion of billings per 100,000 SHI	2011–2015	National Association of Statutory Health Insurance Physicians (KBV)
		ICD-10 C44 (NMSC)			
**Skin cancer prevalence rates**	Outpatient billing data	EBM 01745	Proportion of billings per 100,000 SHI	2011–2015	National Association of Statutory Health Insurance Physicians (KBV)
		EBM 01746			
**Skin cancer mortality rates**	Cause of death statistics	Mortality rate	Number of cases per 100,000 inhabitants	2011–2015	Statistical offices of the federal states (StaLa)
**Socioeconomic and -demographic data**	Indicators and maps of spatial and urban development	Mean age	Years		Federal Office for Building and Regional Planning (BBSR 2022)
		Unemployment rate	%		
		Employment rate	%		
		Household income	Euro		
		Proportion employed without vocational qualification	%		
		County type	1—City regions,		
			2—Regions with urbanisation approaches,		
			3—Rural regions		
		Proportion of foreigners^a^	%		
		Voter turnout^b^	%		
		Proportion of population reaching the nearest dermatologist within 15 minutes	%		Own calculation

***MM*** malignant melanoma, ***NMSC*** non-melanocytic skin cancer. ^a^ People who live in Germany but do not have German citizenship. ^b^ Share of all second votes (valid and invalid) among eligible voters in %.

### Statistical analysis

First, mean values with standard deviation were calculated for each variable and each county over the years 2011–2015. In addition, expected values were calculated using the following formula [15]: ${exp}_{i}=m_{i}∙\frac{\sum_{j=1}^{N}{obs}_{j}}{\sum_{j=1}^{N}m_{j}}$, where m_i_ stands for the number of health insured persons of a county i, $\sum_{j=1}^{N}{obs}_{j}$ for the sum of the number of observed cases and $\sum_{j=1}^{N}m_{j}$ for the sum of the number of health insured persons of all j = 1 to N = 402 counties.

To identify regions with lower or higher screening, prevalence and mortality rates than expected, probability maps were calculated. This procedure detects counties that differ significantly (p < 0.05) from the expected value of a county and illustrates those low and high rate counties on a map. Thus, the statistical significance of the rates and not the rates themselves are used to assign the results. There are three categories: significantly increased (p < 0.05), significantly low (p < 0.05) and not significant (p ≥ 0.05). The counties are coloured in the map according to the category. This was used to test our hypotheses that counties with high screening would have high prevalence and low mortality, and counties with high prevalence would have higher mortality and vice versa. Based on our research questions, six scenarios (A.I–C.II) emerge (Table 2).

**Table 2:** Scenarios A.I–C.II which were differentiated for the analyses.

Scenario	I	II
**A**	High screening–high prevalence	Low screening–low prevalence
**B**	High screening–low mortality	Low screening–high mortality
**C**	High mortality–high prevalence	Low mortality–low prevalence

To identify, to which extent our hypotheses were met, the rate of overlapping counties within these six scenarios were calculated. This was done by dividing the number of counties meeting both criteria for a scenario by the sum of counties meeting only one of the two criteria. As shown in Table 2, the criteria could be high or low prevalence, screening or mortality. The method of overlaps is intended to take an alternative approach. This results from the fact that a spatial-logistic regression has been applied before [7], but its results have left questions open. With this approach, not all counties should be considered, but only selected regions of interest. Regions of interest are based on the three research questions formulated at the beginning. For instance, these regions include those where screening rates are high and skin cancer mortality is low (due to early detection). Conversely, screening rates should be low where skin cancer mortality is high.

For scenarios with at least 5% overlapping rates, a logistic regression was conducted to explain the group classification. Therefore, the counties were classified into groups based on our scenarios: A county was assigned to group 1, if both criteria within the scenario were simultaneously fulfilled, otherwise into group 0. Scenario A.I as an example: If a county has a significantly high value in screening and at the same time a significantly high value in prevalence, this county is assigned to group 1, otherwise (e.g., high screening, low prevalence) to group 0.

Here, the determinants listed in Table 1 were included in the model as explanatory variables. First, the full model was calculated and then backward selection of variables was performed. The Akaike information criterion (AIC) was used to select the model with the highest model goodness of fit. According to AIC, the best model fit is the one that explains the greatest extent of variation using the fewest possible independent variables.

All analyses were performed with R version 3.5.1 (R Foundation, Vienna, Austria). To create the maps we used the R package spdep [16]. A p-value smaller than 0.05 was assumed to be statistically significant. The maps were created with QGIS, version 3.16 (QGIS Development Team, Hannover, Germany).

## Results

An annual average of N = 1,290,213 skin cancer diagnoses and N = 7,534,581 skin cancer screenings were documented nationwide in the SHI system in the years 2011 to 2015. In the same period, an annual average of N = 4,541.2 persons died of skin cancer.

**Fig 1:**
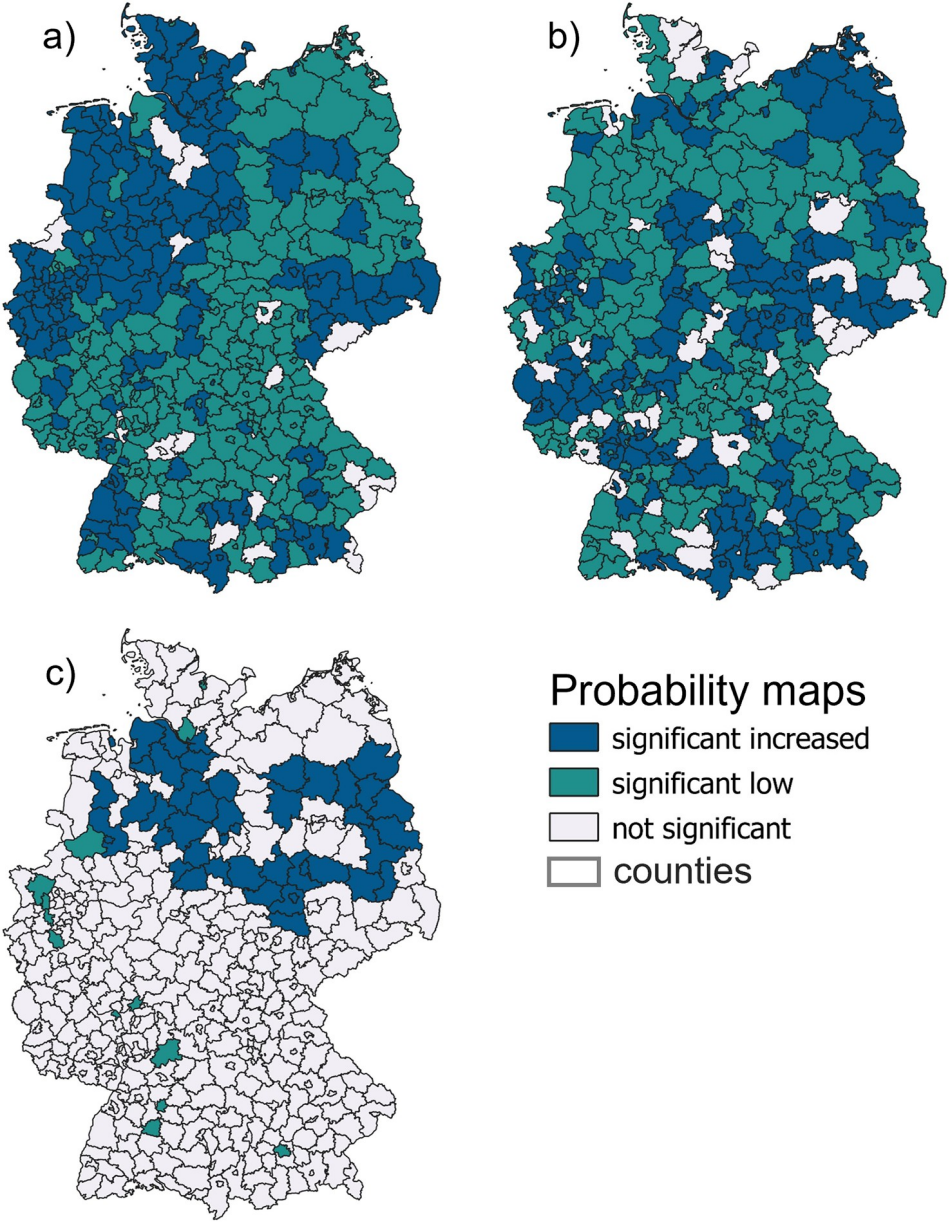
Probability maps on county level of a) skin cancer screening, b) skin cancer prevalence and c) mortality from skin cancer.

For skin cancer screening (Fig 1A), significant increased (p < 0.05, blue) participation rates were observed in particular in the west and north-west of Germany. The remaining counties show significantly decreased (p < 0.05, green) values, except for some counties not diverging from expected average. For skin cancer prevalence (Fig 1B), significantly increased values were detected in the north-east, in the south and in the centre of Germany. Most other regions show significantly decreased values. In terms of skin cancer mortality (Fig 1C), the majority of Germany does not significantly deviate from the expected values. However, in North Germany, a significantly increased mortality rate can be observed.

In the next step, the overlaps of the scenarios from Table 2 were determined. Due to the small (< 5%) overlap, scenario B.I and C.II are omitted here.

### Counties with overlap of a high screening rate and high prevalence rate (A.I) / a low screening rate and low prevalence rate (A.II)

In scenario A.I (high screening–high prevalence), there is an overlap of 69 out of 321 significant counties (21.5%). The remaining 81 counties out of 402 neither have a significantly higher screening rate nor a higher prevalence rate. For socioeconomic and -demographic factors, we found (overlapping counties) a higher unemployment rate, household income and proportion of foreigners for group 1 than for group 0 (all other counties) (Table 3).

**Table 3:** Means and standard deviations of socioeconomic and -demographic factors of counties that have overlap compared to the rest of the counties within the different scenarios. Group 1, both criteria within the scenario were simultaneously fulfilled; group 0, the criteria were not fulfilled.

Scenario	Group	Mean age (years)	Unemployment rate (%)	Employment rate (%)	Household income (€)	Proportion employed without vocational qualification (%)	County type (%)	Proportion of population reaching the nearest dermatologist within 15 min (%)	Proportion of foreigners (%)	Voter turnout (%)
**A.I: High screening–high prevalence **	1	44.5 (1.7)	5.7 (2.5)	59.9 (3.5)	1919.4 (266.7)	11.4 (2.8)	2.20 (0.88)	0.91 (0.14)	10.86 (5.34)	75.9 (3.78)
	0	44.5 (2.0)	5.3 (2.4)	60.0 (4.4)	1862.5 (202.5)	11.8 (3.0)	2.67 (1.05)	0.81 (0.20)	9.86 (5.09)	74.9 (3.78)
**A.II: Low screening–low prevalence **	1	44.3 (2.0)	5.1 (2.5)	60.3 (4.8)	1850.1 (183.3)	12.0 (2.9)	2.74 (1.10)	0.79 (0.21)	10.42 (5.78)	74.7 (3.86)
	0	44.6 (1.9)	5.5 (2.4)	59.8 (4.0)	1880.9 (226.7)	11.6 (3.1)	2.53 (1.01)	0.84 (0.18)	9.88 (4.88)	75.3 (3.75)
**B.II: Low screening–high mortality **	1	47.3 (2.3)	8.1 (1.9)	61.1 (3.3)	1666.6 (73.9)	8.4 (2.6)	3.16 (1.07)	0.66 (0.27)	5.54 (3.92)	70.4 (3.78)
	0	44.4(1.8)	5.2 (2.4)	59.9 (4.3)	1882.4 (215.3)	11.9 (2.9)	2.56 (1.03)	0.83 (0.18)	10.25 (5.10)	75.3 (3.64)
**C.I: High mortality–high prevalence **	1	46.7 (2.0)	7.6 (2.2)	61.0 (3.4)	1722.4 (159.3)	8.4 (2.9)	2.87 (1.06)	0.74 (0.22)	6.3 (3.11)	71.6 (4.3)
	0	44.4 (1.9)	5.2 (2.4)	59.9 (4.3)	1881.3(215.4)	11.9 (2.9)	2.58 (1.04)	0.83 (0.19)	10.26 (5.16)	75.3 (3.7)

The logistic regression (Table 4) shows significant positive associations with the unemployment rate (p = 0.006), the mean age (p = 0.009), the proportion of the population reaching the nearest dermatologist within 15 minutes (p = 0.006) and the voter turnout (p < 0.001).

**Table 4:** Results of logistic regression per scenario to typify the group classification.

Variable	A.I		A.II		B.II		C.I	
	Estimate	p value	Estimate	p value	Estimate	p value	Estimate	p value
(Intercept)	**-45.83**	**7.11e-07**	**13.30**	**0.0004**	6.43	0.62	-1.89	0.06
Mean age	**0.31**	**0.009**	**-0.32**	**0.0002**	0.39	0.08	-	-
Unemployment rate	**0.33**	**0.00577**	**-**	**-**	-	-	**0.31**	**0.001**
Employment rate	0.09	0.09	**-**	**-**	-0.14	0.10	**-**	**-**
Household income	-	-	**-0.003**	**0.007**	-	-	**-**	**-**
Proportion employed without vocational qualification	-	-	**-**	**-**	**-0.32**	**0.03**	**-0.28**	**6.18e-05**
County type					-0.57	0.14	**-**	**-**
Proportion of foreigners	0.08	0.06	**0.09**	**0.002**	-	-	**-**	**-**
Voter turnout	**0.26**	**7.81e-05**	**-0.10**	**0.04**	**-0.17**	**0.04**	**-**	**-**
Proportion of population reaching the nearest dermatologist within 15 minutes	**3.13**	**0.006**	**-1.60**	**0.04**	-2.51	0.07	-	-

*Significant values are shown in* ***bold*** *type*. *(A*.*I*: *High screening–high prevalence*, *A*.*II*: *Low screening–low prevalence*, *B*.*II*: *Low screening–high mortality*, *C*.*I*: *High mortality–high prevalence)*

**Fig 2:**
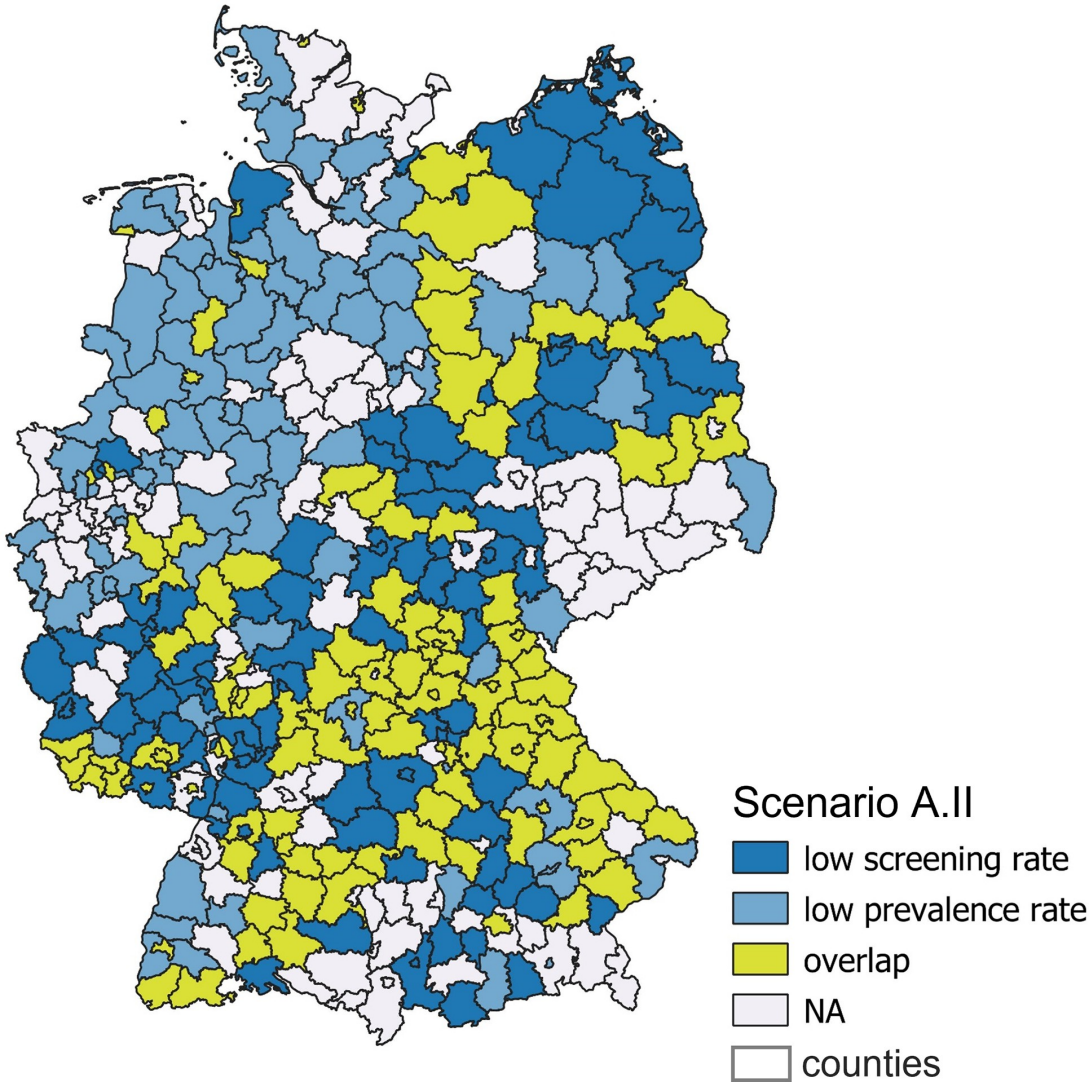
Overlap of counties with a low screening rate and low prevalence rate (A.II).

A total of 113 of 408 counties (27.7%) fall into the overlap of scenario A.II (Fig 2). Counties meeting both criteria are coloured in yellow. The proportion of foreigners is higher in the group with high screening and high prevalence rates (10.42%) than in the rest of the counties (9.88%). On the other hand, the proportion of the population reaching the nearest dermatologist within 15 minutes is lower (0.79% versus 0.84%) (Table 3). Logistic regression shows a positive association with the proportion of foreigners (p = 0.002) and negative associations with the unemployment rate (p < 0.001), household income (p = 0.007), the proportion of the population reaching the nearest dermatologist within 15 minutes (p = 0.02) and voter turnout (p = 0.04) (Table 4).

### Counties with overlap of low screening rate and high mortality rate (B.II)

In a total of 19 out of 256 counties (7.4%) had both, a lower skin cancer screening rate and a higher mortality rate were observed (B.II). These counties showed a higher mean age (47.3 years vs. 44.4 years), higher unemployment rate (8.1% vs. 5.2%), lower household income (1666.6 € vs. 1882.4 €) and lower employed without a professional degree (8.4% vs. 11.9%). The proportion of population reaching the nearest dermatologist within 15 minutes is lower (0.66 vs. 0.83), the proportion of foreigners is lower (5.54% vs. 10.25%) and the voter turnout is lower (70.4% vs. 75.3%) (Table 3). The results of the logistic regression show that the mean age, the employment rate, the proportion of employees without a professional degree, the county type, the proportion of the population that reaches the nearest dermatologist within 15 minutes and the voter turnout provide an explanatory value for this model. Statistically significant are the proportion of employees without a professional degree (p = 0.03) and voter turnout (p = 0.04) (Table 4).

### Counties with overlap of high prevalence and high mortality rate (C.I)

**Fig 3:**
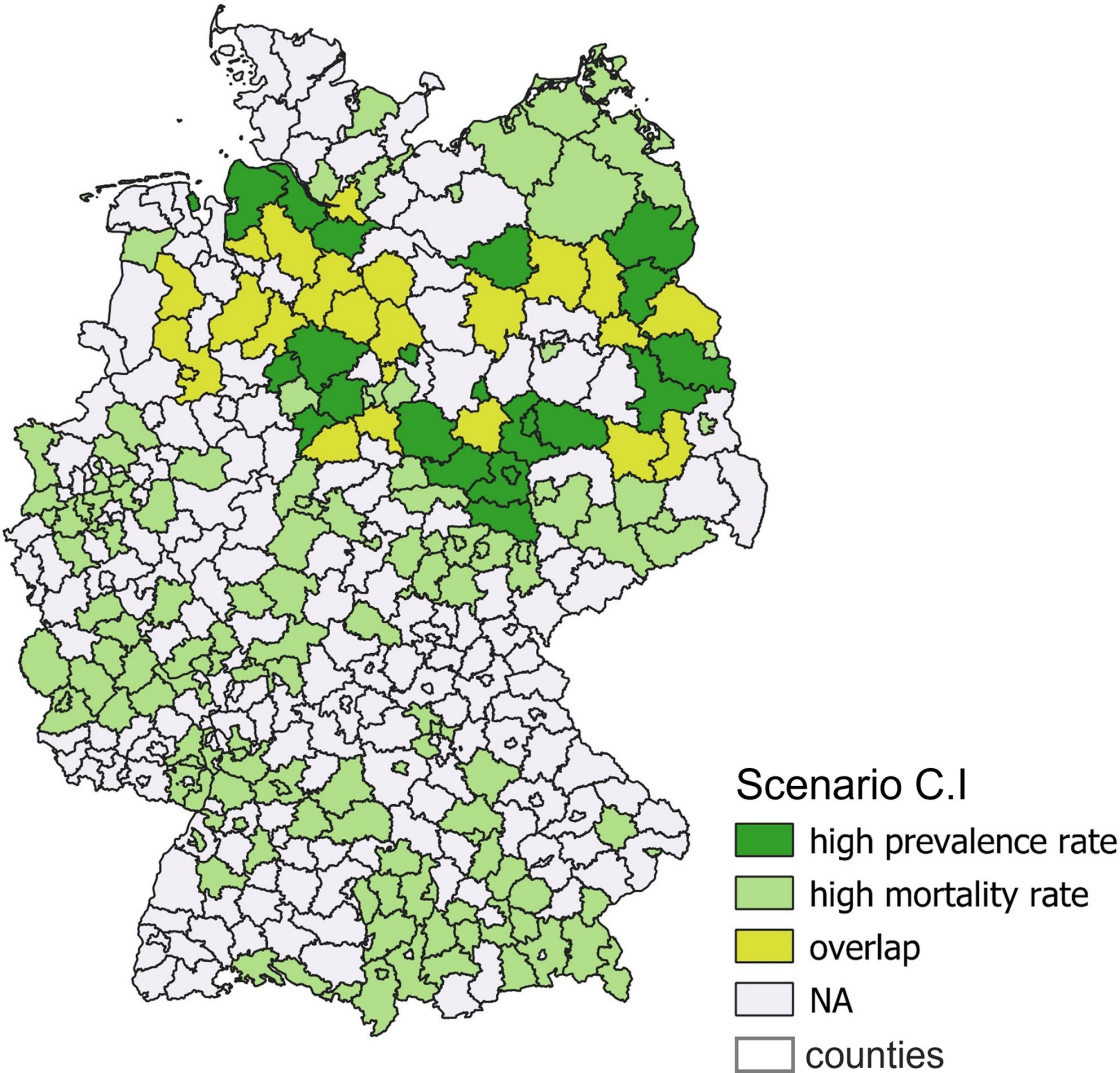
Overlap of counties with high prevalence and high mortality rate (C.1).

In scenario C.I (high mortality–high prevalence), 23 out of 201 (11.4%) overlapped. Fig 3 shows overlapping counties coloured in yellow. The counties with a high prevalence rate only are coloured in light green, and those with a high mortality rate are coloured in dark green.

Compared to the other counties, the overlapping counties of high mortality and high prevalence show lower household income (1722.4 € vs. 1881.3 €), lower proportion of people employed without vocational qualification (8.4% vs. 11.9%), proportion of population reaching the nearest dermatologist within 15 minutes (0.74% vs. 0.83%), proportion of foreigners (6.3% vs. 10.3%) and voter turnout (71.6% vs. 75.3%) (Table 3). According to the logistic regression model, only the unemployment rate (p = 0.001) and the proportion of employees without a professional degree (p < 0.001) provide explanatory value (Table 4).

## Discussion

Studies have identified regional differences in skin cancer screening [9], prevalence [4] and mortality [7] in Germany and other countries (Cecconi et al., Italy [17]; Clarke et al., California [18]; Cancer Research UK, UK [19]). This prompts to question if and how skin cancer screening, prevalence and mortality are associated from a spatial perspective. The aim of this study was therefore to examine the association between screening and prevalence, screening and mortality and mortality and prevalence from a spatial perspective in more detail. To be able to characterise the regions with high or low utilisation of skin cancer screening, prevalence and mortality, relevant socioeconomic and -graphical factors such as educational level, income and accessibility of a dermatologist were obtained from the literature.

Data analyses indicate that the screening rates as well as the skin cancer prevalence and the mortality rates show regional differences. This is in line with publications by Carsin et al. and Ray et al. for prevalence [8, 12] as well as by Augustin et al. (2020) and Vogt et al. for the screening rates [5, 9]. In contrast to the regional variation in morbidity and screening, there are almost no comparable studies on mortality. Cancer Research UK and the Australian Institute of Health and Welfare only describe the mortality caused by skin cancer in a very rough regionalised manner and do not provide any information on the causes and consequences [19, 20].

With regard to spatial associations, not all scenarios that would have supported a possible hypothesis resulted in a sufficiently high number of overlapping districts. In the scenario A.I (high screening rates–high prevalence rates), counties with high screening and prevalence rates are characterised in particular by a higher unemployment rate, household income and voter turnout as well as a higher proportion of the population that can reach the nearest dermatologist within 15 minutes. These results seem plausible, as more screening increases the prevalence (due to more detected tumours). This is also consistent with better access to the nearest physician. The seemingly contradictory association with unemployment cannot be explained, especially as income and voter turnout are also higher in these regions. Counties with low screening and prevalence rates (A.II) are characterised by a higher proportion of employed without vocational qualification and a higher proportion of foreigners. These correlations, i.e. primarily the influence of sociodemographic conditions on skin cancer prevalence and uptake of screening examinations, were also reported in studies by Anastasiadou et al., Akinlotan et al. and Augustin et al. (2018, 2020) [4, 5, 11, 21]. The counties with significantly lower screening and higher mortality rates (B.II) are characterised by an overall lower socioeconomic status: Those employed without a vocational qualification and voter turnout are significantly lower than in the other counties. This suggests that a lower socioeconomic status is associated with less screenings, which could be reflected in higher mortality. In the counties with low mortality and prevalence rates, unemployment rates are higher and the number of employees with vocational qualifications is lower than in the other counties, which also indicates a low socioeconomic status. The results are basically in line with those of scenarios A.I and A.II and also with the results of other studies. This shows, at least in general, that regions with a high use of screening are associated with higher prevalence rates and a better social situation. As far as mortality is concerned, there is also an association with the social situation and possibly also indirectly with the use of screening.

Studies on regional skin cancer mortality are lacking, so no comparison can be made. However, the association with the sociodemographic status seems plausible and may also be indirectly reflected by the regional mortality from skin cancer. Sociodemographic status can be seen as a kind of proxy for individual awareness and thus also health behaviour. Knowledge about the necessity and implementation of early detection examinations is an example of the importance of education. If this knowledge is not available, it can affect both the incidence of skin cancer due to a lack of prevention and mortality due to the lack of early detection. Drexler et al. studied the spatial association of prevalence and mortality for malignant melanoma in Bavaria, Germany [6]. They found a positive correlation between the density of dermatologists with the prevalence. However, the local prevalence could not explain the mortality, i.e. there was also no significant correlation between prevalence and mortality. A further study has shown an association between the region (urban or rural) and the tumour penetration depth in BCC. This tended to be higher in rural areas than in urban areas [22]. This is presumably due to regional differences in the use of screening examinations, which in turn may be associated with the early detection of skin tumours and ultimately also mortality.

All in all, the (spatial) associations between screening, prevalence and mortality are complex and cannot be readily explained. In all cases, socioeconomic and -demographic conditions (e.g., education) are of particular importance. Health literacy and health behaviour are closely correlated with social status and determine, for example, the individual prevention (primary, secondary) of skin cancer. There is also UV radiation, the intensity of which also varies spatially. However, UV has not been taken into account in this study because it is very complex to identify the influence of UV with such a study design, and may be overlaid by the influence of socio-demographic factors, for example [4]. The question about the benefit of screening cannot and should not be answered here.

It should also be mentioned that screening can lead to overdiagnosis–melanomas are discovered through screening that would otherwise not have been detected and would not have led to mortality. This would not support our hypothesis that the mortality rate is low where there is a lot of screening and a high prevalence. It should also be mentioned that the data reflects the screenings billed. It cannot be ruled out that in some cases more screenings were utilised and thus billed than the insured person is entitled to every two years. Age adjustment is important for cancer and cancer mortality. In our case the data were provided to us in aggregated form for three age groups (0–35, 35–65, over 65). A more detailed breakdown was not possible for data protection reasons. As the statutory entitlement to skin cancer screening only exists from the age of 35, we actually only had two age groups available. An age standardisation would not have made sense, but we included the average age of the population as a variable in the analysis in order to be able to take age into account at all.

The study’s strength is its extensive data base for screening and skin cancer prevalence with all people covered by SHI. The substantial amount of additional data on mortality and socioeconomic and -demographical circumstances is another strength. The use of technologies such as GIS to study associations between parameters such as those in this study and geographic location provides valuable information to help decision makers design interventions and process location information [23]. Existing scientific evidence confirms the benefits of such technologies in public health [24–26].

The study has limitations. One limitation is that the mortality pertains to the total population (82 million inhabitants in Germany) and not to those with SHI. This leads to a slight inaccuracy in this study’s comparison of prevalence and screening with mortality. In addition, it should be mentioned that although the methodological approach is suitable to reveal spatial associations of screening, prevalence and mortality, the spatial overlap was rather small in some cases and thus the significance of the results is reduced. In addition, the case numbers of mortality per county are sometimes small, which somewhat reduces the validity of the results. It should also be mentioned that statistical significance is associated with sample size, so that misclassification can occur in counties with a relatively small number of health-insured people but high rate of screening, prevalence or mortality. In addition, we were only able to include dermatologists in the accessibility variable because no location information was available for general practitioners. However, this is unlikely to make too much of a difference to the results, especially as dermatologists carry out more examinations in relative terms [5]. It is also important to note that not all factors influencing skin cancer (skin type) or skin cancer screening (ethnicity) could be taken into account. In this study, we have tried to take into account at least the most important ones. Finally, it is important to note that an ecological fallacy may exist due to the use of spatially aggregated data. No conclusions can be drawn about an individual from the results.

## Conclusions

The aim of the study was to characterize the association between skin cancer screening, prevalence, and mortality from a spatial perspective by only regarding regions of interest. The first statistically significant associations were found between the intersections of the above characteristics. The results indicate that certain socioeconomic and -demographic characteristics play a key role. Overall, however, it can be summarized that this approach does not allow detailed conclusions on the associations between skin cancer screening, prevalence and mortality.
